# Clinical Significance of Early-Onset Alzheimer’s Mutations in Asian and Western Populations: A Scoping Review

**DOI:** 10.3390/genes16030345

**Published:** 2025-03-17

**Authors:** Prevathe Poniah, Aswir Abdul Rashed, Julaina Abdul Jalil, Ernie Zuraida Ali

**Affiliations:** 1 Inborn Errors of Metabolism and Genetics Unit, Nutrition, Metabolic and Cardiovascular Research Centre, Institute for Medical Research, National Institutes of Health, Ministry of Health Malaysia, Setia Alam 41700, Selangor, Malaysia; julaina.jalil@moh.gov.my (J.A.J.); ernie@moh.gov.my (E.Z.A.); 2 Clinical Research Centre, Hospital Raja Permaisuri Bainun, Institute for Clinical Research, National Institutes of Health, Ministry of Health Malaysia, Ipoh 30450, Perak, Malaysia; 3 Nutrition Unit, Nutrition, Metabolic and Cardiovascular Research Centre, Institute for Medical Research, National Institutes of Health, Ministry of Health Malaysia, Shah Alam 41700, Selangor, Malaysia; aswir@moh.gov.my

**Keywords:** early-onset Alzheimer’s disease, mutation, *APP*, *PSEN1*, *PSEN2*, clinical

## Abstract

Background/Objectives: Background: Early-onset Alzheimer’s disease (EOAD) is primarily inherited in an autosomal dominant pattern, with mutations in the *APP*, *PSEN1*, and *PSEN2* genes being central contributors. Diagnosing Alzheimer’s poses challenges due to the coexistence of various co-pathologies, and treatment options remain limited for most patients, apart from familial cases linked to specific genetic mutations. While significant research on Alzheimer’s genetics has been conducted in both Asian and Caucasian populations, the specific mutations and their clinical impacts in EOAD are still inadequately explored. This review aims to provide a detailed analysis of commonly reported genetic mutations and associated clinical features in EOAD patients from Asian and Western populations. Methods: Following the PRISMA-ScR guidelines, a systematic database search was conducted for studies published between 2016 and 2023. After screening 491 records, 36 studies from Asian cohorts and 40 from Western cohorts met the inclusion criteria. Results: The analysis revealed 127 unique mutations in the Asian population and 190 in the Western population. About 16.7% of Asian and 21.9% of Western studies covered both familial and sporadic AD, with consistent patterns across groups. Some mutations were shared between the populations and displayed similar clinical features, while others were population-specific. Conclusions: These findings underscore the considerable variability in EOAD mutations and phenotypes, emphasizing the importance of genetic testing in younger patients to enhance diagnostic accuracy and guide treatment strategies effectively.

## 1. Introduction

Alzheimer’s disease (AD), the most prevalent cause of dementia, is a neurodegenerative disorder that impairs memory and other cognitive functions and is characterized by the accumulation of amyloid-β peptides and hyperphosphorylated tau protein in the brain [1]. The number of people with dementia is expected to reach almost 78 million by 2030, with much of the increase in developing countries [2]. The fastest-growing elderly population is in China, India, and other South Asian countries. As far as the economic impact is concerned, the annual global cost of dementia is now above USD 1.3 trillion and is expected to rise to USD 2.8 trillion by 2030. At present, an estimated 6.7 million Americans aged 65 and older are living with Alzheimer’s dementia [3]. According to Alzheimer’s Disease International, the estimated number of people with dementia (PWD) in Malaysia was 123,000 in 2015. This number was projected to be 261,000 by 2030 and further increase to 590,000 by 2050 [4]. Based on the “Malaysian National Health and Morbidity Survey 2018: Elderly Health”, the overall prevalence of probable dementia was 8.5% (95% CI 6.97 to 10.22) [5].

AD can be categorized into two major types, early-onset AD (EOAD) and late-onset AD (LOAD). EOAD is usually inherited autosomal dominant and occurs before 60–65 years, whereas in LOAD, symptoms typically emerge after the age of 65. Unlike LOAD, which involves significant hippocampal and medial temporal lobe atrophy affecting episodic memory, EOAD often presents with behavioral, visual, or language variants, with memory largely preserved until later stages [6]. A key difference between EOAD and LOAD diagnosis is genetics: EOAD is linked to *APP*, *PSEN1*, and *PSEN2* mutations, while LOAD is primarily associated with the APOE ε4 allele as a risk factor [7]. Patients with EOAD may have behavioral (frontal), visual (posterior cortical atrophy), or language (logopenic) variants with relatively well-preserved memory until the disease progresses [6]. According to the few epidemiologic studies on EOAD, the vast majority are non-familial, accounting for approximately 5% to 10% of all AD. Among these patients, 5% carry pathogenic mutations in one of the AD genes; amyloid precursor protein (*APP*), and presenilin 1 and 2 (*PSEN1* and *PSEN2*).

The *APP* gene is located on chromosome 21q21.3 [8]. The *APP* gene is necessary for physiological processes like synaptogenesis, neural migration, differentiation, proliferation, and plasticity [9]. In AD, APP proteins are known to produce the amyloid-β (Aβ) peptides after proteolytic cleavage. These Aβ peptides are the main component of amyloid plaques in AD. Aβ production is implicated in neurotoxicity and neuronal cell deaths in AD. There are currently about 50 pathogenic *APP* mutations (https://www.alzforum.org/mutations/app, accessed on 19 December 2024). Most of these mutations alter *APP* proteolysis so that A1-42 levels are different from those of other A isoforms [10]. Unlike missense mutations, which have near-complete disease penetrance, *APP* genomic duplications are uncommon and have higher variability in age of onset. A study involving 20 EOAD French families identified 20 *APP* mutations and 5 *APP* duplications [11]. Among these mutations, the c.2149G>A, p. (Val717Ile) substitution present in 12 subjects from 11 families was associated with clinical features of typical AD with amnestic presentation. Mutations at codons 716–717 enhance the production and secretion of Aβ42, which disrupts synaptic signaling and results in early cognitive symptoms, including memory loss [12].

The *PSEN1* gene is another common cause of autosomal dominant EOAD. *PSEN1* mutations are responsible for 70% to 80% of autosomal dominant EOAD cases [13]. The *PSEN1* gene is one of the four core proteins in the γ-secretase complex, which is believed to play a crucial role in the production of amyloid-β (Aβ) from *APP* [14]. Research has shown that hippocampal neurons lacking *PSEN1* significantly reduce γ-secretase activity compared to wild-type neurons [15]. It is expressed in developing neurons during early differentiation stages and is typically found in the cell body; however, punctate labeling indicates dispersion in unidentified cytoplasmic vesicles [16]. Individuals with *PSEN1* mutations experience symptoms 8.4 years earlier than those with *APP* mutations (on average 42.9 years vs. 51.3 years) and 14.2 years earlier than those with *PSEN2* mutations (on average 57.1 years [17]). Similar to *APP*, mutations in the promoter region of *PSEN1* have been associated with an increased risk of EOAD, possibly due to altered *PSEN1* gene expression affecting the Aβ load [18]. Seizures and myoclonus are common symptoms of autosomal dominant EOAD linked to *PSEN1* gene mutation [19]. The specific extent of genotype alteration that links *PSEN1* mutations to seizures in Alzheimer’s disease (AD) remains unclear. It is hypothesized that factors such as an amino acid change, the codon’s position, or the exon involved may play a role.

Within a year of identifying *PSEN1*, another gene encoding the transmembrane protein *PSEN2* was shown to have a substantial connection with Alzheimer’s disease. *PSEN2*’s more restricted localization contributes to the intracellular pool, previously linked to an early event in AD [20]. Presenilin loss has been linked to neuroinflammation and neurodegeneration [21]. In knockout microglial cells, *PSEN2* might boost Aβ-induced classical pro-inflammatory cytokines like IL-1, IL-1, and TNF-α [22]. Compared to the *PSEN1* gene, mutations in the *PSEN2* gene are relatively uncommon. *PSEN2* has fewer than 40 mutations and may increase the activity of γ-secretase. *PSEN2* pathogenic mutations cause a significant decrease in extracellular Aβ40 and Aβ42, and a drastic increase in the Aβ42/40 ratios. Compared to families with *PSEN1* mutations, familial AD with *PSEN2* mutations has a later onset and a more prolonged illness duration [23]. The disease penetrance in AD patients with *PSEN2* mutations is varied, and the onset age ranges from 40 to 80 years old [24]. Only 17 of the 38 mutations are anticipated to cause illness. The pathogenicity of ten mutations is unknown, while T122P, N141I, M239I, and M239V mutations are known to increase Aβ42 levels [25]. Mutations in *PSEN2* disrupt the γ-secretase-mediated cleavage of *APP* into Aβ fragments, leading to a higher Aβ1–42-to-Aβ1–40 ratio, which may arise from increased Aβ1–42 production, reduced Aβ1–40 production, or a combination of these effects.

Racial disparities in molecular biomarkers for AD may indicate race-dependent biological pathways. Although potential racial differences in AD have been investigated, particularly when African Americans are compared to non-Hispanic whites, the evidence is somewhat contradictory [26]. In Asia, EOAD was predicted to have 0.003 instances per 100,000 people in China. In Japan, the estimated prevalence was 0.06 cases/100,000 people, while in South Korea, it was 0.02 cases/100,000 population. Compared to Caucasians, Asian patients had a longer disease duration (median 11 years vs. 8 years, *p* = 0.03) [27]. Nevertheless, studies comparing the differences in mutations and the clinical outcomes in Asians and Caucasians are scarce. The available ones warrant future studies in larger cohorts due to their small samples. Aside from that, these studies only looked at a few variants, and other mutations are likely to have been discovered in recent years. Therefore, previous studies may not be comprehensive due to publication bias [27].

More than 230 mutations in one of these three genes have been identified in EOAD patients. These mutations increase amyloid-β 42 production, resulting in an earlier onset of AD. Identifying EOAD is critical, as the family should receive genetic counseling. Furthermore, identifying the underlying mutations contributes to understanding the pathophysiology of EOAD. Most significantly, asymptomatic carriers of mutations may be perfect candidates for future clinical trials of disease-modifying treatments for AD. However, there is little evidence in the literature to support the relevance of these mutations in EOAD clinical manifestations. Currently, there is no effective treatment for AD, but potential therapeutic solutions might be successful in the early stages of the disease. Hence, further research is needed to unravel the role of genetic mutations underlying unexplained EOAD, both familial and sporadic. Over the years, considerable advances have been made in characterizing the genes and genetic variants involved in this disease. Still, the genetic factors identified so far account only for a portion of the underlying genetic basis of disease. Thus, we are far from having a complete understanding of the genetic architecture of AD. To explore this gap in the literature, we conducted a scoping review to investigate the most prominent mutations within *APP*, *PSEN1*, and *PSEN2* genes in EOAD and their clinical significance in Asian and Western regions.

## 2. Materials and Methods

### 2.1. Search Strategy

Original articles were searched in four databases (PubMed, Scopus, Wiley, and ScienceDirect) and one search engine, Google Scholar, from January 2016 to December 2023 using the Medical Subject Heading (MeSH) terms “early-onset Alzheimer’s disease”, crossed with the terms “mutation” AND “clinical” AND “*APP* gene” AND “*PSEN1* gene” AND “*PSEN2* gene “. Publications with available abstracts were reviewed, and our search was limited to studies published in English only. Papers on human and clinical trials related to Alzheimer’s were included. However, review articles, proceedings, and letters to the editor studies were excluded, and duplicate articles were eliminated. All articles were reported in line with PRISMA Extension for Scoping Reviews (PRISMA-ScR) [28].

### 2.2. Study Selection

A pair of authors independently assessed the titles and abstracts during the initial screening. Differences in the initial assessment were resolved by a discussion leading to a consensus, with a third party serving as arbitrator when necessary. The full texts that were appropriate and included in the review following the initial abstract screening were read by two authors. Each study was recorded as included, excluded, or unclear. Full articles were retrieved for further assessment if recorded as included or unclear. Any disagreement was re-evaluated and re-assessed among the reviewers.

### 2.3. Sampling Design

#### 2.3.1. Inclusion Criteria

Published intervention studies (defined as a randomized controlled trial, crossover study, and quasi-experimental study) on *APP*, *PSEN1*, and *PSEN2* mutations and their clinical association in EOAD patients were included. We included only human studies with adult participants aged at least 18 years old from both genders. Studies were included if they analyzed at least one of the genetic markers mentioned earlier.

#### 2.3.2. Exclusion Criteria

The exclusion criteria were as follows:

(a) Types of articles other than original research papers, such as abstracts, review papers, conference proceedings, book or book chapters;
(b) Articles that were not in the English language;
(c) Articles published before January 2016; we opted to review articles published from 2016 onwards because the MeSH terms used in our search showed a higher number of publications beginning in that year, coinciding with the introduction of next-generation sequencing technologies. Additionally, the primary focus of this review was to compare genetic findings in EOAD between Asian and Western cohorts;
(d) Studies on mutations other than *APP*, *PSEN1*, or *PSEN*;
(e) Alzheimer’s research solely focused on late-onset AD, as this would not reflect EOAD;
(f) Articles concerning dementia other than those related to Alzheimer’s (other forms/causes of dementia, i.e., Lewy body, vascular, frontotemporal, Parkinson’s, Huntington’s).

### 2.4. Identifying Research Questions and Relevant Studies

During the identification process, relevant English-language articles were explored. Considering the research questions, the literature search of articles was guided by PICO: ‘P’ population (people with EOAD), ‘I’ intervention/phenomenon of interest (mutations in *APP*, *PSEN1*, and *PSEN2* genes), and ‘C’ comparison/context (biochemical markers) and ‘O’ outcome (clinical outcome). The keywords that were used to describe the gene mutations and their clinical implications in EOAD were used to construct the strategies. The Boolean operator, ‘AND’ was used during the search process.

### 2.5. Assessing Studies for Eligibility

The Preferred Reporting Items for Systematic Reviews and Meta-Analysis Extension for Scoping Reviews (PRISMA-ScR) framework was used in this study for the review process [28]. All potential papers (title, author’s names, and year of publication) were managed on an MS Excel spreadsheet during the search strategy phase. Titles throughout the abstract screening process were used to vet all potential papers, removing duplicates or irrelevant articles. To organize the data, a PRISMA flow diagram was used. The two investigators independently analyzed the full-text publications to see if they matched the inclusion/exclusion criteria in the second step. Any discordant full-text papers were re-evaluated, and any remaining issues about research eligibility were resolved through conversation with a third investigator until a full consensus was reached.

### 2.6. Data Analysis

Articles for reading were distributed to all researchers equitably involved in the study. For each article, all identified mutations from the selected genes were tabulated. The research team created a data collection instrument (MS Excel) to confirm study relevance and extract study characteristics, which included questions about the research proposal [type of publication, language, country, and year; and the article (journal, title, methodology, gene details such as exon, mutation, and amino acid changes, in-silico analysis, results, and conclusions)]. In our scoping review, we described key categories such as target populations; key mutations within the *APP*, *PSEN1*, and *PSEN2* genes; clinical significance; and family history. In addition, we incorporated pathogenicity prediction (based on in silico, in vitro, or in vivo models) as well.

## 3. Results

### 3.1. Selection of Studies

**Figure 1:**
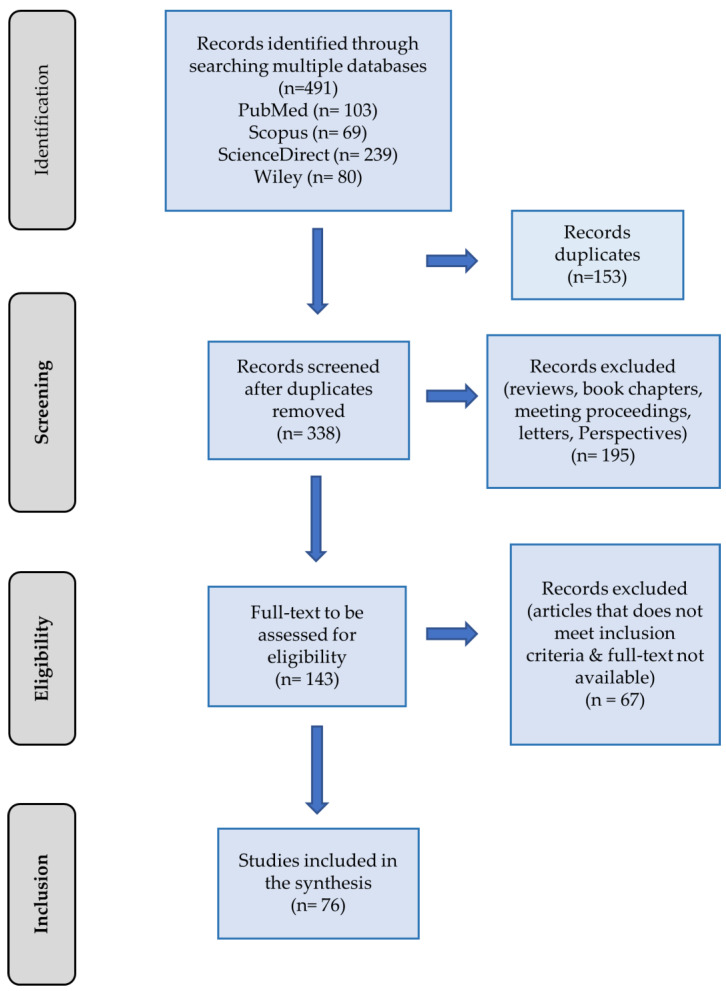
PRISMA flowchart depicting the study selection process for inclusion in the review.

A comprehensive search across four electronic databases and one search engine yielded a total of 491 articles. After eliminating duplicates, 338 unique articles remained for screening. Additional refinement of the studies involved excluding records categorized as reviews, book chapters, meeting proceedings, letters, and perspectives. This process yielded 143 full texts eligible for assessment. Applying the inclusion criteria narrowed down the selection to 76 articles for inclusion in this review. The entire flow of study selection, from identification to inclusion, along with the articles identified at each stage, is illustrated in Figure 1.

### 3.2. Characteristics of the Studies

**Figure 2:**
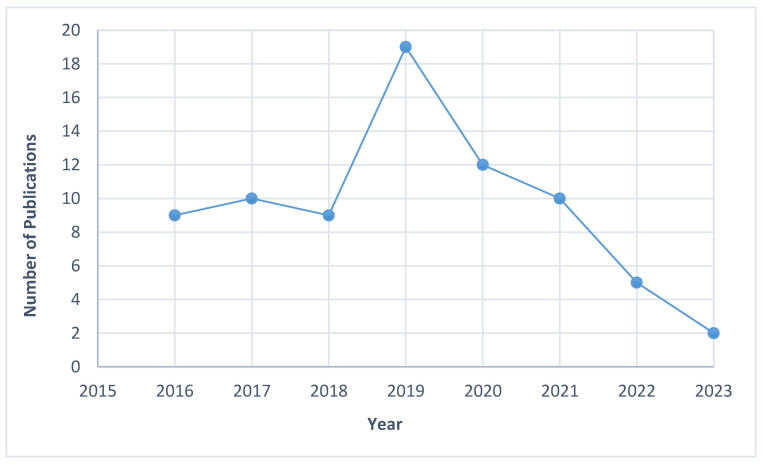
Distribution of studies published between 2016 and 2023.

Figure 2 and Table 1 displays the overall features of the studies incorporated within this review. The proportion of publications detailing the genetic aspects of EOAD increased consistently between 2016 and 2019. Conversely, 2020 to 2023 witnessed a notable decline in the number of studies, with only approximately 9.2% of EOAD investigations focusing on *APP*, *PSEN1*, and *PSEN2* genes reported between 2022 and 2023. Out of the 76 studies examined in detail, more studies originated from the Western region compared to Asia. Specifically, 52.6% of the studies were conducted in the West, while the rest focused on Asian populations.

Studies conducted in Asia exhibited a comparable distribution of case reports and cross-sectional studies, whereas Western studies predominantly encompassed cross-sectional studies (approximately 47.5%), followed by cohort studies, at around 25%. The EOAD, also known as familial Alzheimer’s disease (FAD), is characterized by its hereditary nature. Hence, a considerable portion of studies were either familial or a combination of familial and sporadic cases. Both Asian and Western publications reported relatively fewer studies on sporadic EOAD, comprising only about 2–7% of the total studies. Around 5% of studies from both regions did not specify the EOAD history. Overall, the characteristics of EOAD publications, particularly those focusing on the genetics of *APP*, *PSEN1*, and *PSEN2*, were comparable between the two regions, with a similar number of publications in each category.

### 3.3. Common Mutations Identified in Asian and Western Populations

**Figure 3:**
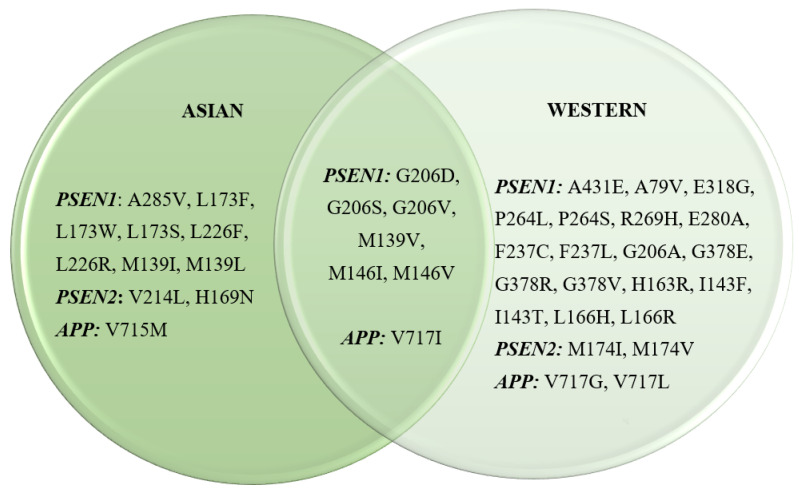
Most prominent EOAD mutations in Asian and Western countries.

A combined total of 127 mutations within the *APP*, *PSEN1*, or *PSEN2* genes were detected across 36 publications originating from Asian sources. Among these, eight mutations within *PSEN1*, two within *PSEN2*, and one within *APP* were exclusively identified in Asian populations. Conversely, among the 190 mutations compiled from 40 publications, not of Asian origin, 18 mutations within *PSEN1*, 2 within *PSEN2*, and 2 within *APP* were unique to Western populations. Notably, there were six mutations within *PSEN1* and one within *APP* that were observed in both Asian and Western populations (Figure 3). Tables S1 and S2 provide detailed descriptions of these mutations, including insights into patient demographics, familial histories, and the clinical significance associated with these genetic variations. Overall, most publications documenting genetic mutations in *APP*, *PSEN1*, and *PSEN2* align with previous research, indicating the autosomal dominant nature of EOAD. This review underscores this observation, with 70.1% of the studies reporting mutations in patients with strong familial histories, suggesting a predominantly familial nature of EOAD. Approximately 16.7% (Asian) and 21.9% (Western) of studies reported findings from both familial and sporadic AD, while the rest were either sporadic or were not mentioned. This distribution remained consistent across both Asian and Western populations.

### 3.4. Distribution of APP, PSEN1, and PSEN2 Mutations in Asian and Western Populations

Mutations were predominantly distributed across Asian countries, primarily originating from China, Korea, Taiwan, Iran, and South Korea, with China exhibiting the highest mutation count, followed by Korea. In Western countries, mutations were observed in a wide range of nations, including Belgium, Brazil, Colombia, Finland, France, Hungary, Italy, Mexico, the Netherlands, Spain, Sweden, the UK, and the USA. Meanwhile, most publications documenting mutations within Western populations focused on findings from the USA, followed by the UK and France.

Among Asian countries, seven publications from China reported the common *APP* mutation V717I to be associated with EOAD. Comparatively, several countries from the Western population had also reported V717I as their prominent *APP* mutation in EOAD patients, amounting to six publications altogether. The next most common mutation is the missense mutation at location 206 from the *PSEN1* gene, causing a change in the amino acid glycine to either alanine, valine, serine, or aspartic acid. These mutations were commonly observed in both populations, with seven publications from findings among Asians and seven studies from the West. Similarly, the change in the amino acid methionine to either valine, lysine, or threonine at codon 139 is another prevalent mutation observed in Asian patients with EOAD. However, only the M139V mutation was identified in the Western EOAD patients, thus becoming the mutation mutually shared in both populations.

**Figure 4:**
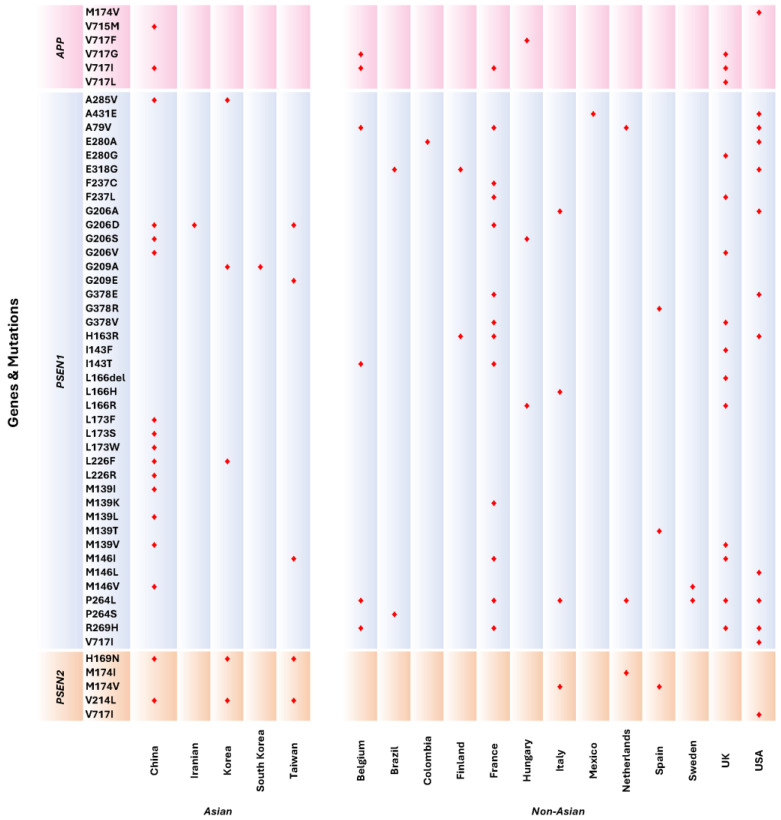
Distribution of mutations according to *APP*, *PSEN1*, and *PSEN2* genes from Asian and Western countries. Markers in red indicate mutations reported according to countries.

It is noteworthy that *APP* and *PSEN1* mutations are more prevalent in EOAD patients, while *PSEN2* has been relatively less observed. There were four studies identified from the Western EOAD population with mutations of the *PSEN2* gene (M174I and M174V). Comparatively, more Asian studies have identified two *PSEN2* mutations, with six studies reporting the H169N mutation and six publications reporting the V214L mutations. Three of these studies [29,30,31] have mentioned either one or both *PSEN2* mutations in their publications (Figure 4).

### 3.5. Clinical Phenotypes for Shared Mutations Observed in Both Asian and Western Populations

Several mutations were found in both the Asian and Western cohorts. These mutations were from the *PSEN1* (G206D, G206S, G206V, M139V, and M146V) and *APP* (V717I) genes. Some of these mutations exhibited similar clinical characteristics such as memory impairment and behavioral change, while other clinical symptoms were observed in one population but not in the other. Patients with the G206D, G206S, M139V, M146V, and V717I showed similar clinical presentations in both the Asian and Western populations. However, patients with the Gly206Val mutation found in *PSEN1* displayed differences in both populations; patients from the Asian population were more likely to present with memory loss and irritation or anxiety, while AD patients from the Western population were found to experience neurological changes that result in myoclonus followed by seizures. Another mutation that showed differences in the clinical phenotype was the Met146Ile mutation. This missense mutation, in which hydrophobic methionine is replaced by the amino acid isoleucine, resulted in isolated progressive cognitive decline in the Western population. In contrast, AD patients from Asia with this mutation experience typical amnestic symptoms along with more severe neurological conditions, such as seizures and other extrapyramidal symptoms, while also having emotional lability (Table 2).

## 4. Discussion

The findings summarized in this study provide an overview of the published literature investigating the clinical presentation of patients having mutations in the three most prominent EOAD-related genes: *APP*, *PSEN1*, and *PSEN2*. Although the disease pathophysiology is similar, there have been differences in the clinical phenotypes of the patients who harbor these gene mutations. Alzheimer’s disease that manifests before the age of 65, known as EOAD, though slightly more prevalent than cases of familial Alzheimer’s disease (FAD), comprises less than 5% of pathologically diagnosed Alzheimer’s cases [2]. Previous studies have pointed out various types of AD pathophysiology such as cerebrospinal fluid levels of amyloid-β (Aβ) [32] and other clinical presentations such as cognitive impairments with executive dysfunction and disorientation, language impairment, and memory loss in patients with mutations of either *APP*, *PSEN1*, or *PSEN2* genes. However, no studies have directly compared the clinical outcomes of individuals with mutations in these genes, especially considering the differences between the Asian and Western populations. This review, therefore, was performed to gain more insights into the effects of *APP*, *PSEN1*, and *PSEN2* gene mutations in patients diagnosed with EOAD from both cohorts. This study’s evidence could shed light on the different mutations and how they may contribute towards more effective treatment and management of patients with EOAD.

Among the three common genes responsible for EOAD, *PSEN1* was most prominently observed, with more than 360 mutations reported worldwide in the Alzforum database (https://www.alzforum.org/mutations/psen-1, accessed on 19 December 2024), followed by the *APP* and *PSEN2* gene mutations. The *PSEN1* gene encodes presenilin-1, a subunit of γ-secretase, the aspartyl protease responsible for Aβ generation. Mutations in *PSEN1* may result in an excess production of amyloid-β 1–42 and the accumulation of amyloid deposits in the brain [33]. The review’s findings indicate that the highest number of *PSEN1* mutations in the Asian region were reported from China, while most of these gene mutations in the Western world were identified in France, the UK, and the USA. In a study involving 148 probands from unrelated families in Mainland China, 8 *PSEN1* variants were identified among 65 EOAD cases [34].

A highly common mutation site of *PSEN1* in Asian patients was residue 139. A novel *PSEN1* mutation was reported: NM_000021.3; c415A>T, p.(Met139Leu). Subsequently, the same mutation was also reported in other Chinese populations [35,36,37]. While the clinical implications of this mutation were not available for all studies mentioned above, one of the studies associated M139L with memory decline as well as sensory and movement disorders in a female EOAD patient who had a positive family history [35]. Another mutation causing an amino acid change from methionine to valine (M139V) at residue 139 resulted in a slightly different clinical phenotype, wherein language impairment and mental and behavioral changes were observed in a patient with the mutation, unlike M139L, which suggests that phenotypic variations persist even with the same codon site due to differences in amino acid transversions. As such, genetic mutations may represent a paradigm in EOAD and are worth considering as a valuable component in disease diagnosis. In addition to M139L and M139V, there was another mutation identified at this site, M139I, present in Chinese patients with familial Alzheimer’s disease [38], which was also previously reported in Korean patients [39]. Patients from both populations had early memory impairment without anynon-cognitive neurological features.

All mutations at residue 139 in Asians were found in familial EOAD. In the Western population, however, this mutation was seen in both sporadic as well as familial EOAD. There were also two other amino acid changes found at this site among Western populations, M139K and M139T, which were absent in the Asian individuals [11,40,41]. These patients were exhibiting symptoms such as typical amnestic symptoms and isolated progressive cognitive decline. The clinical characteristics evident in individuals with this mutation appeared to be more pronounced compared to those observed in Asian patients, including symptoms like seizures and spastic paraparesis. Mutations at residue 139 were reported in several studies from China; however, there were no other Asian studies that reported this mutation, suggestive of a causative variant specific to the South Chinese population. This finding is consistent with the findings published by Jiao et al. [35].

Variants at residue 206 were collectively identified as another prominent *PSEN1* mutation site in both Asian and Western populations. Asian patients with the G206D mutation exhibited memory impairment [35,42] [43], while isolated progressive cognitive decline was observed in French patients. While the G206D mutation is more common in the Asian cohort, the G206A mutation is prevalent in Western populations and has not been observed in Asians. According to a study by Bartoletti-Stella et al. [44], all patients demonstrated evidence of an AD pathophysiological process, as indicated by characteristic AD CSF biomarkers. Additionally, other amino acid changes at this site, such as G206S and G206V variants, were found in both populations. In a familial study by Li et al. [33], the G206V mutation was reported as de novo and suggested as a potential causative mutation in EOAD patients. This mutation was found in a 34-year-old man with slowly progressing memory loss and anxiety, with functional imaging showing bilateral temporal lobe and hippocampal atrophy.

In the Western cohort, the *PSEN1* P264L missense mutation is another common variant found in familial EOAD cases. However, this mutation has also been identified in sporadic cases in some studies [44,45]. Several in silico algorithms have predicted that the amino acid substitution from proline to leucine at codon site 264 is damaging, which is associated with its clinical presentation and neuropathology in EOAD patients [46]. Notably, this variant has not been reported in Asian EOAD cases, indicating that it may be specific to the Western population. Patients with this mutation often experience memory impairment, behavioral changes, or spastic paraparesis [11,47]. An in vivo study using mice models showed that the *PSEN1* P264L/P264L double gene-targeted mice had accelerated levels of Aβ42, leading to Aβ deposition [48]. Elevated Aβ42 levels are known to increase hippocampal atrophy rates in mild cognitive impairment [49]. Although this is just one of many mutations that could contribute to EOAD, its strong association with Alzheimer’s pathology suggests that further investigation into this variant could be crucial for developing targeted treatments for Alzheimer’s patients.

Although less commonly involved compared to *PSEN1*, another hotspot site in EOAD is the *PSEN2* gene. In this review, the widely known pathological H169N missense mutation appears to be most prevalent in the Asian population. This variant was initially discovered in two unrelated Chinese individuals [50]. In addition to its association with LOAD, the *PSEN2* His169Asn mutation was linked to frontotemporal dementia in this study. Giau et al. [51] also reported this mutation in the Korean population, where it was observed in both sporadic and familial AD cases, similar to the Chinese patients. Additionally, this variant was found in another Han Chinese patient with a family history of LOAD. A common clinical presentation among patients in these studies was memory loss [51,52]. In a 63-year-old female Korean patient, there was also noticeable cognitive decline and language impairment. This patient scored 0 on the Korean Mini-Mental State Examination (K-MMSE) and had a Clinical Dementia Rating (CDR) score of 3, indicating severe dementia [53]. Interestingly, this mutation was not found in the Western population, suggesting that it is unique to East Asian ancestry and warrants further investigation.

The *APP* gene is another significant pathogenic variant implicated in AD. Currently, there are approximately 114 mutations listed for AD in the Alzforum database (https://www.alzforum.org/mutations/app), highlighting the diverse genetic landscape of the disease. Among these mutations, the amino acid codon 717 is particularly well documented in autosomal dominant EOAD [11,54]. The missense mutation V717I, resulting in a valine to isoleucine substitution, is a notable example shared by both Asian and Western populations. Patients with the V717I mutation exhibit heterogeneous clinical manifestations, including dementia, language impairment, neuropsychiatric symptoms, cerebellar ataxia, spastic paraparesis, and schizophrenic-like syndromes [38,55]. This mutation has thus far only been identified in familial AD cases in both populations, indicating a strong association with autosomal dominant inheritance. In addition to the V717I mutation, Western patients have also been reported to carry the V717G and V717L variants [6,32,56]. A study investigating the distribution of amyloid-β (Aβ) pathologies across different cortical layers in familial AD (FAD) mutation cases analyzed brain tissues from 20 FAD cases. This study found cerebral amyloid angiopathy in two cases with the *APP* mutation (specifically V717 and V717L) [56]. These findings underscore the importance of genetic testing in AD cases due to its significant role in AD pathophysiology. Identifying pathologically important variants of EOAD genes could lead to earlier detection of the disease and facilitate a more targeted treatment approach. This, in turn, could improve the prognosis and quality of life for AD patients and their caregivers.

Secondly, while several mutations were present in both populations, many other mutations were specific to each population across all three genes. The clinical features also varied among these mutations. This is noteworthy because validated information on variant-specific clinicopathological outcomes could be useful for clinicians in the differential diagnosis of EOAD patients, leading to a more targeted approach in treatment intervention. Having substantial genetic epidemiology data for a population allows for the provision of genetic counseling in due course, especially since early intervention improves outcomes in neurodegenerative disorders. Patients could then opt for genetic counseling and have the opportunity to participate in clinical trials aimed at delaying or preventing the onset of symptoms. However, some limitations of this review should be noted. Several publications included in this review lack detailed clinical phenotype information. Many of these studies focus on discussing the biological relevance of the mutations and their implications in terms of imaging findings, with scarce data provided on the clinical symptoms or significance. Furthermore, we initially incorporated data on biochemical markers during the charting process. However, due to incomplete information on this parameter and limited details on their association with gene mutations, we ultimately had to exclude biochemical markers from the overall analysis for the review. Therefore, our review may not be comprehensive due to publication bias. We also did not report or discuss the in silico findings of mutations specific to EOAD, as the scope of this review focused solely on clinical findings. Further investigation into the in silico results of EOAD variants could add value by enhancing our understanding of the biological and clinical relevance of these mutations. This would be extremely helpful in providing a complete picture of the pathophysiology of EOAD.

## 5. Conclusions

As such, while our review presents a valuable comparison of both populations regarding the genetic makeup of EOAD, it should be interpreted with caution. The absence of detailed clinical information and additional findings associated with the pathogenic variants means our conclusions are preliminary and warrant further investigation. Future studies on Alzheimer’s disease (AD) should place emphasis on exploring biochemical markers such as Aβ and tau proteins. Their significance lies in providing a deeper understanding of the diverse phenotypes of AD and potentially elucidating the disease’s pathophysiology when linked to genetic mutations. In addition, leveraging in silico approaches helps facilitate the virtual screening of therapeutic compounds, enabling the identification of candidates that may prevent or slow the progression of early AD without the need for extensive in vitro or in vivo testing initially. In conclusion, this review highlights the importance of investigating genetic mutations in EOAD, as this could inform the best treatment options for each patient group, considering that no one-size-fits-all approach exists. Early intervention in AD could significantly improve the quality of life for patients and their families.

## Figures and Tables

**Table 1:** Characteristics of studies included in the full-text review (n = 76).

Study Characteristics	Number of Studies (n)	Percentage (%)
**Countries**		
Asian	36	47.4
Western	40	52.6
**Type of Study**		
***Asian***		
Case Report	11	30.6
Brief Communication	3	8.3
Case–Control	4	11.1
Cohort	6	16.7
Cross-Sectional	12	33.3
***Western***		
Case Report	8	20
Brief Communication	0	0
Case–Control	3	7.5
Cohort	10	25
Cross-Sectional	19	47.5
**EOAD History**		
***Asian***		
Familial	27	75
Sporadic	1	2.8
Familial and Sporadic	6	16.7
Unknown	2	5.5
***Western***		
Familial	26	65
Sporadic	3	7.5
Familial and Sporadic	9	22.5
Unknown	2	5

**Table 2:** Clinical phenotypes for mutually present mutations in Asian and Western populations.

Mutations Mutually Present in Asian and Western Populations	Clinical Phenotype (Asian)	Clinical Phenotype (Western)
**G206D**	memory decline, mental and behavioral change	isolated progressive cognitive decline
**G206S**	earlier mean AAO, severe cognitive impairment	memory impairment, disorientation, hallucination, psychotic sessions, conversion, mixed dissociative disorder, myoclonus, impaired speech, and apraxia
**G206V**	slowly progressing memory loss combined with irritation and anxiety	myoclonus, seizures
**M139V**	memory decline, language impairment, mental and behavioral change	behavioral presentation, myoclonus, seizures, spastic paraparesis with/without other pyramidal signs
**M146I**	myoclonus, seizure, extrapyramidal symptoms, emotional lability	isolated progressive cognitive decline
**M146V**	memory decline, language impairment, mental and behavioral change	Slight short-term memory dysfunction and spatial disorientation, myoclonic epileptic seizures, and gait difficulties.
**V717I**	memory decline, language impairment, mental and behavioral change	typical of AD with amnestic presentation

## Data Availability

The original contributions presented in the study are included in the article/Supplementary Material, further inquiries can be directed to the corresponding author.

## Supplementary Materials

The following supporting information can be downloaded at: https://www.mdpi.com/article/10.3390/genes16030345/s1 [6,11,13,29,30,31,32,33,34,35,36,37,38,40,41,42,43,44,45,47,52,55,56,57,58,59,60,61,62,63,64,65,66,67,68,69,70,71,72,73,74,75]. Table S1: Common mutations among Asian population; Table S2: Common mutations among Western population.
