# Supplementary material for: Clinical Significance of Early-Onset Alzheimer’s Mutations in Asian and Western Populations: A Scoping Review
Source: Genes (Basel). 2025 Mar 17;16(3):345. doi: 10.3390/genes16030345 (PMC11942072; doi:10.3390/genes16030345)
Supplement: Supplementary file 1 [file genes-16-00345-s001.zip › genes-3411048-supplementary.pdf]

Table S1: Common mutation among Asian population

Gene	Protein change	Nucleotide change	Country	AOO (Years)	Family history (Familial or Sporadic)	Clinical diagnosis	References
APP	V715M		China		Familial	*No details available*	Quan M et al., 2020 [37]
APP	V715M	G/A	China	42	Familial	*No details available*	Jia L et al., 2020 [71]
APP	V715M	c.2143G>A	China	51	Familial	memory decline, mental & behavioural change	Jiao B et al., 2021 [35]
APP	V717I	c.2149G>A	China	>44	Familial	cognitive impairments with executive dysfunction and disorientation	Zhang G et al., 2017 [72]
APP	V717I	c.2149G>A			Familial	memory deficits, occasional seizures, and swallowing problems	Jiang B et al., 2019 [38]
APP	V717I	c.2149G>A			Familial	*No details available*	Gao Y et al., 2019 [34]
APP	V717I	c.2149G>A			Familial	*No details available*	Zhou J et al., 2020 [73]
APP	V717I		China		Familial	*No details available*	Quan M et al., 2020 [37]
APP	V717I	G/A	China	61	Familial	*No details available*	Jia L et al., 2020 [71]
APP	V717I	c.2149G>A	China	47	Familial	memory decline, language impairment, mental & behavioural change	Jiao B et al., 2021 [35]
PSEN1	A285V	c.854C > T	Korea	46	Familial/Sporadic	progressive memory dysfunction	Giau VV et al., 2019 [13]
PSEN1	A285V		China		Familial	*No details available*	Quan M et al., 2020 [37]
PSEN1	A285V	G/A	China	65	Familial	*No details available*	Jia L et al., 2020 [71]
PSEN1	A285V	c.854C>T	China	46	Familial	memory decline, language impairment,	Jiao B et al., 2021 [35]
PSEN1	G206D	c.617G>A	Iranian	33-45	Familial	Memory loss	Wang JC et al., 2017 [42]
PSEN1	G206D	c.617G>A		33	Sporadic	memory impairment, seizures	Lin YS et al., 2020 [43]
PSEN1	G206D	c.617G>A	China	38	Familial	memory decline, mental & behavioural change	Jiao B et al., 2021 [35]
PSEN1	G206D	c.G617A			Familial	*No details available*	Hsu JL et al., 2021 [29]
PSEN1	G206S		China		Familial	increased fiber numbers of putamen-rMFG circuit, increased diffusivity of the left hippocampus-PCC circuit and volume reduction of all regions	Quan M et al., 2020 [37]

PSEN1	G206S	G/A	China	37	Familial	earlier mean AAO, severe cognitive impairment	Jia L et al., 2020 [71]
PSEN1	G206V	c.641A>G	China	30	Sporadic	slowly progressing memory loss combined with irritation and anxiety	Li YS et al., 2019 [33]
PSEN1	G209A	c.626G>C	South Korea	40	Familial	mild cognitive impairment with depression& progressive memory decline	An SS et al., 2016 [74]
PSEN1	G209A	c.626G > C	Korea		Familial	*No details available*	Giau VV et al., 2019 [31]
PSEN1	G209A	c.626 G > C	Korea	54	Probably	*No details available*	Giau VV et al., 2019 [31]
PSEN1	G209E	c.626G>A		60	Familial	*No details available*	Lin YS et al., 2020 [43]
PSEN1	L173F		China		Familial	*No details available*	Quan M et al., 2020 [37]
PSEN1	L173F	G/C	China	38	Familial	*No details available*	Jia L et al., 2020 [71]
PSEN1	L173F	c.519G>T	China	37	Familial	memory decline, mental & behavioural change	Jiao B et al., 2021 [35]
PSEN1	L173S	T/C	China	38	Familial	*No details available*	Wang G et al., 2019 [42]
PSEN1	L173S	T/C	China	36	Familial	*No details available*	Jia L et al., 2020 [71]
PSEN1	L173W	c.518T>G			Familial	*No details available*	Jiang B et al., 2019 [38]
PSEN1	L226F	c.676C>T		37	Sporadic	Memory loss	An SS et al., 2016 [74]
PSEN1	L226F	c.676C>T			Familial	*No details available*	Gao Y et al., 2019 [34]
PSEN1	L226F	CTC > TTC	Korea		Familial	*No details available*	Giau VV et al., 2019 [13]
PSEN1	L226R	c.677T>G	China	60	Familial	Language impairment	Ma L et al., 2019 [52]
PSEN1	L226R	c.677T>G	China	44	Familial	memory decline, mental & behavioural change	Jiao B et al., 2021 [35]
PSEN1	M139I	c.417G>A			Familial	Early memory impairment. Non-cognitive neurological feature absent	Jiang B et al., 2019 [38]
PSEN1	M139L	c.415A>T	China		Familial	typical amnestic symptoms	Gao Y et al., 2019 [34]
PSEN1	M139L	c.415A>T	China	56	Familial	Memory impairment; visuospatial dysfunction; personality changes; agitation; abnormal behaviours; disinhibition; myoclonus.	Qiu Q et al., 2019 [36]
PSEN1	M139L		China		Familial	*No details available*	Quan M et al., 2020 [37]
PSEN1	M139L	A/T	China	45	Familial	*No details available*	Jia L et al., 2020 [71]

PSEN1	M139L	c.415A>T	China	38	Familial	memory decline, sensory and movement disorders	Jiao B et al., 2021 [35]
PSEN1	M139V		China		Familial	*No details available*	Quan M et al., 2020 [37]
PSEN1	M139V	A/G	China	51	Familial	*No details available*	Jia L et al., 2020 [71]
PSEN1	M139V	c.415A>G	China	53	Familial	memory decline, language impairment, mental & behavioural change	Jiao B et al., 2021 [35]
PSEN1	M146I	c.438G>T		44.7	Familial	Typical amnestic symptom, seizures, jerks, myoclonus extrapyramidal symptoms, emotional lability	Lin YS et al., 2020 [43]
PSEN1	M146I	c.G438A			Familial	*No details available*	Hsu JL et al., 2021 [29]
PSEN1	M146V	A/G	China	36	Familial	*No details available*	Jia L et al., 2020 [71]
PSEN1	M146V	c.436A>G	China	42	Familial	memory decline, language impairment, mental & behavioural change	Jiao B et al., 2021 [35]
PSEN2	H169N	c.505C>A	Korea	50	Sporadic	Memory loss	Giau VV et al., 2018 [51]
PSEN2	H169N	c.505C > A	Korea		Familial	*No details available*	Giau VV et al., 2019 [13]
PSEN2	H169N	c.505 C > A	Korea	59	Unknown	*No details available*	Giau VV et al., 2019 [13]
PSEN2	H169N	c.505C>A	China	63	Familial	Memory loss	Ma L et al., 2019 [52]
PSEN2	H169N	c.C505A			Familial	*No details available*	Hsu JL et al., 2021 [29]
PSEN2	H169N	C/A	China		Familial	*No details available*	Xie XY et al., 2021 [75]
PSEN2	H169N	c.505C>A	China	50	Familial	Progressive memory decline, language disability	Liang Z et al., 2023 [30]
PSEN2	V214L	c.640G>T		54	Familial	Memory loss, anomia	An SS et al., 2016 [74]
PSEN2	V214L	c.640G > A			Familial	*No details available*	Giau VV et al., 2019 [13]
PSEN2	V214L		China		Familial	*No details available*	Quan M et al., 2020 [37]
PSEN2	V214L	G/T	China	42	Familial	*No details available*	Jia L et al., 2020 [71]
PSEN2	V214L	c.G640T			Familial	*No details available*	Hsu JL et al., 2021 [29]
PSEN2	V214L	c.640G>T	China	47	Familial	Memory disturbance	Liang Z et al., 2023 [30]

Table S2: Common mutation among Western population

Gene	Protein Change	Nucleotide Change	Country	AOO (years)	Family History (Familial or Sporadic)	Clinical diagnosis	References
APP	M174V	g.6206A>G	USA	54	Familial/ Sporadic	*No details available*	Wingo TS et al., 2019 [45]
APP	V717F	c.2149G>T	Hungary	40	Familial	Severe short-term memory impairment; serious language impairment, epileptic seizure, myoclonus-like jerks, hypokinesis	Csaban D et al., 2022 [57]
APP	V717G	c.2150T>G	Belgium		Familial	*No details available*	Perrone F et al., 2020 [32]
APP	V717G	c.2150T>G	UK	50	Familial	*No details available*	Ryan NS et al., 2016 [6]
APP	V717I	c.2149G>A	UK	49	Familial	CAA were observed in the frontal cortices	Willumsen N et al., 2022 [56]
APP	V717I	c.2149G>A	Belgium	58	Familial	*No details available*	Perrone F et al., 2020 [32]
APP	V717I	NA	USA	36	Familial	The association between generalization and left hippocampal volumes persisted in the gene mutation carriers only and not in non- carrying kin.	Petok JR et al., 2018 [58]
APP	V717I	c.2149G>A	UK	52	Familial	*No details available*	Ryan NS et al., 2016 [6]
APP	V717I	g.275341G>A	USA		Familial/ Sporadic	*No details available*	Wingo TS et al., 2019 [45]
APP	V717I	c.2149G>A	France	40	Familial	typical of AD with amnestic presentation	Lanoiselée HM et al., 2017 [11]
APP	V717L	c.2149G>C	UK	51	Familial	CAA were observed in the frontal cortices	Willumsen N et al., 2022 [56]
APP	V717L	c.2149G>C	UK	49	Familial	*No details available*	Ryan NS et al., 2016 [6]
PSEN1	A431E	c.1292C>A	Mexico	43	Familial	Memory loss, gait abnormalities, language and visuospatial disorders, seizures, and apraxia.	Dumois-Petersen S et al., 2020 [55]
PSEN1	A431E	g.71096C>A	USA		Familial/ Sporadic	*No details available*	Wingo TS et al., 2019 [45]
PSEN1	A431E		USA	35	Familial	mild developmental delay, chronic night-time behavioural disturbance, and onset of	Parker J et al., 2019 [61]

						progressive cognitive deficits, spastic paraparesis and pseudobulbar affect	
PSEN1	A431E		USA		Familial	*No details available*	Petok JR et al., 2018 [58]
PSEN1	A431E	c.1292C>A	USA	48	Familial	Decreases in fractional anisotropy and increases in mean diffusivity in widespread white-matter areas including the corpus callosum, occipital, parietal, and frontal lobes in PSEN1 mutation carriers with spastic paraparesis.	Soosman SK et al., 2016 [59]
PSEN1	A79V	c.236C>T	Belgium		Familial	*No details available*	Perrone F et al., 2020 [32]
PSEN1	A79V	g.22921C>T	USA		Familial/ Sporadic	*No details available*	Wingo TS et al., 2019 [45]
PSEN1	A79V	c.236C>T	Netherlands	64	Familial	cognitive impairment, delusions, hallucinations, and parkinsonism	Wong TH et al., 2020 [47]
PSEN1	A79V	c.236C>T	France	63	Familial	isolated progressive cognitive decline	Lanoiselée HM et al., 2017 [11]
PSEN1	E280A	c.839A>C	Colombia	30	Familial	cognitively impaired	Quiroz YT et al., 2020 [60]
PSEN1	E280A		USA		Familial	*No details available*	Petok JR et al., 2018 [58]
PSEN1	E280A	c.839A>C	Colombia	30	Familial	gradual memory loss, followed by changes in behavior and language impairment	Tariot PN et al., 2018 [62]
PSEN1	E280G	c.839A>G	UK	42	Familial	*No details available*	Willumsen N et al., 2022 [56]
PSEN1	E280G	c.839A>G	UK	42	Familial	CAA were observed in the frontal cortices, higher Aβ load in lower cortical layers, increased cotton wool plaque	Ryan NS et al., 2016 [6]
PSEN1	E318G	c.A953G	Finland		Familial	*No details available*	Luukkainen L et al., 2019 [63]
PSEN1	E318G	c.A953G	USA		Unknown	*No details available*	N'Songo A et al., 2017 [64]
PSEN1	E318G	c.953A>G	Brazil		Familial/ Sporadic	*No details available*	Abdala BB et al., 2017 [65]
PSEN1	F237C	c.710T>G	France	25	Sporadic	typical	Lacour M et al., 2019 [40]
PSEN1	F237C	c.710T>G	France	25	Sporadic	isolated progressive cognitive decline	Lanoiselée HM et al., 2017 [11]
PSEN1	F237L	c.709T>C	UK	47	Familial	*No details available*	Ryan NS et al., 2016 [6]
PSEN1	F237L	c.711T>A	France	47	Familial	isolated progressive cognitive decline	Lanoiselée HM et al., 2017 [11]
PSEN1	G206A	g.44636G>C	USA		Familial/ Sporadic	*No details available*	Wingo TS et al., 2019 [45]

PSEN1	G206A		USA		Familial	*No details available*	Petok JR et al., 2018 [58]
PSEN1	G206A	c.617G>C	USA		Familial	*No details available*	Soosman SK et al., 2016 [59]
PSEN1	G206A	c.617G > C	Italy	57	Familial/ Sporadic	All patients had evidence of AD pathophysiological process as defined by the presence of a characteristic AD CSF biomarker	Bartoletti-Stella A et al., 2022 [44]
PSEN1	G206D	c.617G>A	France	30	Familial	isolated progressive cognitive decline	Lanoiselée HM et al., 2017 [11]
PSEN1	G206S	c.616G>A	Hungary	39	Sporadic	Memory impairment, disorientation, hallucination, psychotic sessions, conversion, mixed dissociative disorder, myoclonus, impaired speech, and apraxia	Csaban D et al., 2022 [57]
PSEN1	G206V	c.617G>T	UK	30	Familial	*No details available*	Ryan NS et al., 2016 [6]
PSEN1	G378E		USA		Familial	*No details available*	Petok JR et al., 2018 [58]
PSEN1	G378E	c.1133G>A	France	45	Familial	Atypical presentation: cerebellar ataxia and extra pyramidal syndrome.	Lanoiselée HM et al., 2017 [11]
PSEN1	G378R	c.1132G>C	Spain	46	Familial	episodic memory problems, diminished language fluency, and word-finding difficulties	Ramos-Campoy O et al., 2020 [66]
PSEN1	G378V	c.1133G>T	UK	44	Familial	*No details available*	Ryan NS et al., 2016 [6]
PSEN1	G378V	c.1133G>T	France	38	Familial	isolated progressive cognitive decline	Lanoiselée HM et al., 2017 [11]
PSEN1	H163R	c.A488G	Finland	39	Familial	Rapidly progressive cognitive decline, slight postural tremor in her upper limbs, clumsy movements, and myoclonic jerks, euphoric, behavioral disinhibition.	Luukkainen L et al., 2019 [63]
PSEN1	H163R		USA		Familial	*No details available*	Petok JR et al., 2018 [58]
PSEN1	H163R	c.488A>G	France	34	Sporadic	frontal variant, myoclonic seizures	Lacour M et al., 2019 [40]
PSEN1	H163R	c.488A>G	France	34	Familial & Sporadic	isolated progressive cognitive decline	Lanoiselée HM et al., 2017 [11]
PSEN1	I143F	c.427A>T	UK	56	Familial	*No details available*	Ryan NS et al., 2016 [6]
PSEN1	I143T	c.428T>C	Belgium		Familial	*No details available*	Perrone F et al., 2020 [32]
PSEN1	I143T	c.428T>C	France	35	Sporadic	typical, paraparesis	Lacour M et al., 2019 [40]
PSEN1	I143T	c.428T>C	France	35	Sporadic	isolated progressive cognitive decline	Lanoiselée HM et al., 2017 [11]
PSEN1	L166del	c.496_498del	UK	38	Familial	*No details available*	Ryan NS et al., 2016 [6]
PSEN1	L166H	c.497 T>A	Italy		Familial	*No details available*	Perrone F et al., 2018 [68]

PSEN1	L166H	c.497T > A	Italy	57	Familial/ Sporadic	All patients had evidence of AD pathophysiological process as defined by the presence of a characteristic AD CSF biomarker profile	Bartoletti-Stella A et al., 2022 [44]
PSEN1	L166R	c.497T>G	UK	40	Familial	*No details available*	Ryan NS et al., 2016 [6]
PSEN1	L166R	c.497 T>G	Hungary	45	Familial	Spastic paraparesis, dysarthria, dysphagia, severe cognitive decline, and progressive loss of speech	Csaban D et al., 2022 [57]
PSEN1	M139K	c.146T>A	France	37	Sporadic	Typical amnestic	Lacour M et al., 2019 [40]
PSEN1	M139K	c.416T>A	France	37	Sporadic	isolated progressive cognitive decline	Lanoiselée HM et al., 2017 [11]
PSEN1	M139T	c.416T>C	Spain	60	Sporadic	*No details available*	Carmona-Iragui M et al., 2017 [41]
PSEN1	M139V	c.415A>G	UK	40	Familial	*No details available*	Willumsen N et al., 2022 [56]
PSEN1	M139V	c.415A>G	UK		Familial	Behavioural presentation, myoclonus, seizures, spastic paraparesis with/without other pyramidal signs	Ryan NS et al., 2016 [6]
PSEN1	M146I	c.438G>A	France	42	Familial	isolated progressive cognitive decline	Willumsen N et al., 2022 [56]
PSEN1	M146I	c.438G>A	UK	48	Familial	*No details available*	Lanoiselée HM et al., 2017 [11]
PSEN1	M146L	c.436A>C	USA		Familial	*No details available*	Soosman SK et al., 2016 [59]
PSEN1	M146V	c.436A>G	Sweden	34	Sporadic	Slight short term memory dysfunction and spatial disorientation already at about 30 years of age. At later disease stages the patient suffered from myoclonic epileptic seizures and gait diffculties.	Pagnon de la Vega M et al., 2022 [67]
PSEN1	P264L	c.791C>T	Belgium		Familial	*No details available*	Perrone F et al., 2020 [32]
PSEN1	P264L	g.49976C>T	USA		Familial/ Sporadic	*No details available*	Wingo TS et al., 2019 [45]
PSEN1	P264L	c.791C>T	Netherlands	56	Familial	Memory impairment, behavioral changes, and impairment in word comprehension and word findin	Wong TH et al., 2020 [47]
PSEN1	P264L	c.791 C>T	Italy		Familial	*No details available*	Perrone F et al., 2018 [68]
PSEN1	P264L	c.791 C>T	Italy	55	Familial	Probable AD dementia with intermediate evidence of AD pathophysiological process (NIA-AA), Typical AD (IWG-2),	Bartoletti-Stella A et al., 2018 [69]
PSEN1	P264L	c.791C>T	UK	50	Familial	*No details available*	Ryan NS et al., 2016 [6]

PSEN1	P264L	c.791C>T	France	41	Familial	displayed an associated phenotype of spastic paraparesis	Lanoiselée HM et al., 2017 [11]
PSEN1	P264L	c.791C > T	Italy	57	Familial/ Sporadic	Evidence of AD pathophysiological process as defined by the presence of a characteristic	Bartoletti-Stella A et al., 2022 [44]
PSEN1	P264L	c.791C>T	Sweden	42	Familial	global cognitive impairment and early personality changes	Pagnon de la Vega M et al., 2022 [67]
PSEN1	P264S	c.790C>T	Brazil	49	Familial	Amnestic	Takada LT et al., 2022 [70]
PSEN1	R269H	c.806G>A	Belgium		Familial	*No details available*	Perrone F et al., 2020 [32]
PSEN1	R269H	g.49991G>A	USA		Familial/ Sporadic	*No details available*	Wingo TS et al., 2019 [45]
PSEN1	R269H		USA		Familial	*No details available*	Petok JR et al., 2018 [58]
PSEN1	R269H	c.806G>A	UK	55	Familial	*No details available*	Ryan NS et al., 2016 [6]
PSEN1	R269H	c.806G>A	USA		Familial	*No details available*	Soosman SK et al., 2016 [59]
PSEN1	R269H	c.806G>A	France	60	Familial	isolated progressive cognitive decline	Lanoiselée HM et al., 2017 [11]
PSEN1	M146I	c.438G>T	UK	48	Familial	CAA were observed in the frontal cortices	Ryan NS et al., 2016 [6]
PSEN2	M174I	c.522G>A	Netherlands	51	Familial	Memory impairment, myoclonus, seizure, delusions, spasticity, extrapyramidal sign	Wong TH et al., 2020 [47]
PSEN2	M174V	c.520A>G	Spain	50	Sporadic	*No details available*	Ramos-Campoy O et al., 2020 [66]
PSEN2	M174V	c.520A > G	Italy	57	Familial/ Sporadic	All patients had evidence of AD pathophysiological process as defined by the presence of a characteristic AD CSF biomarker profile	Bartoletti-Stella A et al., 2022 [44]
